# Carotid Atheroinflammation Is Associated With Cerebral Small Vessel Disease Severity

**DOI:** 10.3389/fneur.2021.690935

**Published:** 2021-08-31

**Authors:** Nicholas R. Evans, Jason M. Tarkin, Jessica Walsh, Mohammed M. Chowdhury, Andrew J. Patterson, Martin J. Graves, James H. F. Rudd, Elizabeth A. Warburton

**Affiliations:** ^1^Department of Medicine, University of Cambridge, Cambridge, United Kingdom; ^2^Department of Clinical Neurosciences, University of Cambridge, Cambridge, United Kingdom; ^3^Division of Cardiovascular Medicine, University of Cambridge, Cambridge, United Kingdom; ^4^Department of Surgery, University of Cambridge, Cambridge, United Kingdom; ^5^Department of Radiology, University of Cambridge, Cambridge, United Kingdom

**Keywords:** atherosclerosis, blood-brain barrier, carotid artery, cerebrovascular disease/stroke, leukoaraiosis, carotid-cerebrovascular interface

## Abstract

**Background:** Atherosclerosis is a systemic inflammatory disease, with common inflammatory processes implicated in both atheroma vulnerability and blood-brain barrier disruption. This prospective multimodal imaging study aimed to measure directly the association between systemic atheroma inflammation (“atheroinflammation”) and downstream chronic cerebral small vessel disease severity.

**Methods:** Twenty-six individuals with ischemic stroke with ipsilateral carotid artery stenosis of >50% underwent ^18^fluoride-fluorodeoxyglucose-positron emission tomography within 2 weeks of stroke. Small vessel disease severity and white matter hyperintensity volume were assessed using 3-tesla magnetic resonance imaging also within 2 weeks of stroke.

**Results:** Fluorodeoxyglucose uptake was independently associated with more severe small vessel disease (odds ratio 6.18, 95% confidence interval 2.1–18.2, *P* < 0.01 for the non-culprit carotid artery) and larger white matter hyperintensity volumes (coefficient = 14.33 mL, *P* < 0.01 for the non-culprit carotid artery).

**Conclusion:** These proof-of-concept results have important implications for our understanding of the neurovascular interface and potential therapeutic exploitation in the management of systemic atherosclerosis, particularly non-stenotic disease previously considered asymptomatic, in order to reduce the burden of chronic cerebrovascular disease.

## Introduction

Atherosclerosis is a systemic inflammatory disease that may cause stroke through destabilization of atherosclerotic plaques and consequent thromboemboli (1). However, it is increasingly recognized that the effects of atherosclerosis extend beyond a single “vulnerable plaque,” and instead involve the overall burden from the systemic nature of atherosclerosis on the individual “vulnerable patient” (2).

This is particularly true in the neurovascular setting, where the brain represents an end-organ highly sensitive to insult from the general metabolic environment. The presence of vascular risk factors may exacerbate inflammation within atheroma (atheroinflammation) (3–6), disrupt blood-brain barrier (BBB) integrity (7), and promote neuroinflammation in individuals without stroke, potentially priming the brain for injury (8). Furthermore, systemic inflammation itself may also promote an increase in BBB permeability (9). Consequently, chronic pro-inflammatory states, such as that seen in atherosclerosis, may have a role in compromising BBB integrity. Such BBB dysfunction is implicated in the development of chronic cerebral small vessel disease (SVD) (10); focal lacunar infarcts or subcortical diffuse white matter change (leukoaraiosis) characterized by neuronal loss, demyelination, and gliosis (10). SVD is a major risk factor for both stroke and dementia (11), and is independently associated with poorer recovery after stroke (12) and stroke recurrence (13).

The direct relationship between carotid atherosclerosis and SVD remains unclear. Although leukoaraiosis is positively associated with both carotid intima-media thickness (IMT) and presence of atheroma, negative associations have been reported with the degree of luminal stenosis (14). These inconsistent findings may be due to variability in the extent of inflammation within atheroma, which is independent of stenosis severity (15). Inflammation within atheroma can be measured *in vivo* by positron emission tomography (PET) using ^18^fluoride-fluorodeoxyglucose (FDG), a radionuclide analog of glucose (16). FDG uptake is increased in symptomatic carotid atheroma (15), and correlates with histological macrophage density but not plaque size (17).

This study examines the direct association between carotid artery atheroinflammation, measured by FDG-PET/CT, and the severity of cerebral SVD. We hypothesized that increased carotid artery FDG uptake would be associated with more severe leukoaraiosis.

## Materials and Methods

### Participants

The Imaging Carotid Atherosclerosis in the Recovery and Understanding of Stroke Severity (ICARUSS) Study prospectively recruited individuals presenting with an ischemic stroke within the previous seven days due to ipsilateral common or internal carotid artery stenosis of ≥50% measured on computed tomography angiography (CTA) [using the North American Symptomatic Carotid Endarterectomy Trial method (18)] at Addenbrooke's Hospital, Cambridge, United Kingdom. Cardiovascular risk factors and stroke severity were recorded at baseline. Only individuals with evidence of brain infarction on diffusion-weighted imaging (DWI) were enrolled. The minimum age for study eligibility was 40 years. Individuals with atrial fibrillation were excluded.

Anonymized imaging reads were performed for the full study cohort after study completion, with readers (NRE, JMT, JW, MMC) blinded to the clinical data. PET and MRI analyses were analyzed independently and matched with clinical information and each other only after analysis of the full cohort was complete.

All participants provided written informed consent in accordance with the Declaration of Helsinki. The study protocol was approved by a national research ethics committee (Nottingham One Research Ethics Committee, 14/EM/0128).

### PET/CT Protocol

FDG-PET/CT (Discovery 690 GE Healthcare, Little Chalfont, UK) scans were performed with 64-slice computed tomography within 14 days of ischemic stroke. Participants fasted for 6 h prior to injection. Participants were injected intravenously with a target of 250 MBq of FDG (sourced from Erigal Ltd, Keele, UK), followed by a 90-min uptake time, as per previous work (19). A silence protocol (minimal vocalization, only small sips of water permitted) was adopted during this uptake period to reduce physiological tracer uptake in neighboring structures. In participants without diabetes, blood glucose concentrations were confirmed as ≥7.0 mmol/L prior to tracer injection. Participants with diabetes mellitus were instructed to take their usual oral antidiabetic medications as normal, but insulin was omitted within the 4 h prior to imaging.

PET imaging datasets were analyzed using OsiriX (version 5.7.1, OsiriX Imaging Software, Geneva, Switzerland). Co-registered PET and CT images were resampled to 3 mm slice thickness and regions of interest (ROIs) drawn manually on fused PET/CT images along the common carotid and internal carotid artery to encompass the region 0.9 cm proximal and 3 cm distal to the carotid bifurcation as per established methodology (15). ROIs were then transferred onto co-registered PET to produce standardized uptake values of the maximum uptake within the ROI (SUV_max_). To compensate for blood pooling, the tissue SUV was adjusted for venous SUV – the average of mid-luminal ROIs in the jugular vein over five contiguous 3 mm slices without evidence of spill-over from neighboring structures – to give the maximum target-to-background ratio (TBR_max_); a measure of radiotracer uptake validated for use in vascular PET imaging (17).

TBR_max_ for culprit and non-culprit carotid arteries were compared for the most diseased segment (MDS) and whole vessel (WV). The MDS considers the most diseased 9 mm of the artery (based on tracer uptake) and represents the mean of the TBR_max_ of the ROIs in three contiguous axial slices where the central ROI constitutes the point of highest tracer uptake in the artery as per previous methodology (15). The WV is the median of tracer uptake across all 14 axial slices of the artery. An experienced reader (MMC) performed reproducibility and quality assurance by repeating ROIs in 20% of the FDG-PET/CTs.

### MRI Protocol

Participants had brain imaging performed within 2 weeks of stroke using a 3-tesla whole body magnetic resonance imaging (MRI) scanner (MR750, GE Healthcare, Waukesha, WI) with a 12-channel head, neck, and spine coil with a brachial plexus attachment. Sequences included T1, T2, DWI, fluid-attenuated inversion recovery (FLAIR), and gradient echo sequences.

### Assessment of Cerebral Small Vessel Disease

The extent of WMH was measured both semi-quantitatively and quantitatively. Semi-quantitative measures were taken from the FLAIR sequence using the scoring system proposed by Fazekas et al. (20) and later modified by Pantoni et al. (21). The Fazekas score has been dichotomized previously (22), and in this study we dichotomized global (whole brain) periventricular and deep white matter hyperintensities according to no/mild or moderate/severe leukoaraiosis [using the visual scale described by Pantoni et al. (21)] given that the majority of our cohort showed some small vessel disease.

Quantitative measurement of WMHs was performed by measuring WMHs in the hemisphere contralateral to the acute stroke and multiplying by two. Measurement was conducted using semi-automatic ROI marking using Jim Imaging Software (version 7.0, Xinapse Systems Ltd., Essex, United Kingdom).

MRI interpretation was performed by two experienced readers for all scans (NRE and JW). Intra-class correlation coefficients for inter-rater reliability were calculated subsequently.

### Inflammatory Biomarker

Venous blood was drawn at the time of FDG-PET/CT for high-sensitivity C-reactive protein (hsCRP) as a marker of inflammation.

### Statistical Analysis

Continuous data was tested for normality using the Shapiro-Wilk method. Parametric data was reported as mean ± standard deviation (SD) and non-parametric data reported as median and inter-quartile range (IQR). Unpaired groups were compared using *t*-testing (parametric readings) or Wilcoxon rank sum testing (non-parametric readings). Comparison between culprit and contralateral non-culprit arteries in the same individual used equivalent paired testing. Associations were tested using two-tailed Spearman's rho correlation (non-parametric or ordinal data) or Pearson's correlation coefficient (parametric data).

Multivariable analysis (logistic regression and linear regression) initially included all variables considered in univariable analysis (age, sex, smoking status, diabetes mellitus, hypertension, pre-stroke statin, pre-stroke antiplatelet, cardiovascular history), with goodness of fit optimized subsequently with backwards elimination of variables to achieve the lowest Akaike information criteria.

Tracer uptake was compared across stenosis categories (“1–29%,” “30–49,” “50–69%,” “70–89%,” “90–99%”) in both symptomatic and asymptomatic arteries using Kruskal-Wallis one-way ANOVA testing (for non-parametric data).

The cut-off for statistical significance was set at *P* = 0.05. Data was analyzed using R (version 3.6.1, 2019, R Foundation for Statistical Computing, Vienna, Austria).

### Data Availability

The corresponding author had full access to all the data in the study and takes responsibility for its integrity and the data analysis. The full anonymized dataset is available upon reasonable request from the corresponding author.

## Results

### Study Population

Of the 31 participants recruited to the ICARUSS study, 28 underwent FDG-PET/CT (of the three recruited who did not undergo scanning: two deteriorated clinically, becoming too unwell to continue in the study, and one was unable to complete imaging due to claustrophobia).

Of this 28, 26 had imaging suitable for analysis (one participant had an uninterpretable PET scan and one subject declined MRI). All participants had bilateral carotid atherosclerosis. Eight (30.8%) participants had co-existent coronary artery disease, and four (15.4%) had a clinical diagnosis of peripheral arterial disease. Clinical characteristics are shown in Table 1.

**Table 1:** Clinical characteristics of study cohort (*n* = 26).

Mean age (years)	74.8 (SD 9.7)
Men	18 (69.2%)
Median BMI	26 (IQR 3.9)
Smoking history (current or ex-smokers)	17 (65.4%)
Diabetes mellitus	4 (15.4%)
Hypertension	17 (65.4%)
Pre-stroke statin	9 (34.6%)
Pre-stroke antiplatelet	8 (30.8%)
Cardiovascular history (previous ischemic heart disease/ myocardial infarction)	8 (30.8%)
Median National Institutes of Health Stroke Scale (NIHSS)	4.5 (IQR 10.75)
Thrombolysed	6 (23.1%)
Modal degree of symptomatic stenosis	70–89%

All acute infarcts were cortical in their distribution, consistent with probable artery-to-artery embolization. Reflecting this, in all cases the carotid pathology was felt by the clinical team to be the causative etiology for the acute infarct. The median DWI lesion volume was 3.36 ml (IQR 14.4 ml).

### PET Tracer Uptake in Culprit and Non-culprit Atherosclerotic Plaque

FDG uptake was significantly higher in the culprit artery than in the contralateral non-culprit carotid artery for both the MDS [median TBR_max_ (IQR) 2.08 (0.52) vs. 1.89 (0.40), respectively, *P* < 0.001] and WV measures of uptake [median TBR_max_ (IQR) 1.92 (0.41) vs. 1.71 (0.31), respectively, *P* < 0.001]. No relationship was observed between FDG MDS TBR_max_ and the degree of luminal stenosis (*P* = 0.91). There was a moderate association between hsCRP and non-culprit WV TBR_max_ (r_s_ = 0.50, *P* = 0.02). Inter-rater reliability of FDG reads was 0.93.

### Chronic Small Vessel Disease

Of the 26 participants, 15 (57.7%) had no/mild leukoaraiosis, 11 (42.3%) had moderate/severe leukoaraiosis. The pattern of disease was predominantly peri-ventricular. The median WMH volume was 3.11 ml (IQR 7.43 ml). The group with moderate/severe leukoaraiosis were older than those with no/mild leukoaraiosis (mean age 79.4 ± 9.7 vs. 71.5 ± 8.5 years, *P* = 0.04), otherwise there were no other significant differences in clinical characteristics between the cohorts (Table 2).

**Table 2:** Comparison of dichotomized groups of small vessel disease severity.

	**No/Mild** **leukoaraiosis** **(*n* = 15)**	**Moderate/Severe** **leukoaraiosis** **(*n* = 11)**	**Significance**
Mean age (SD) (years)	71.5 (± 8.5)	79.4 (± 9.7)	*P =* 0.04
Number of males (%)	11 (73.3%)	7 (63.3%)	*P =* 0.60
Mean BMI (SD)	26.0 (± 4.2)	28.3 (± 5.3)	*P =* 0.26
Current/former smoker (%)	10 (66.7%)	7 (63.3%)	*P =* 0.87
Diabetes mellitus (%)	1 (6.7%)	3 (27.3%)	*P =* 0.15
Hypertension (%)	11 (73.3%)	6 (54.5%)	*P =* 0.32
Pre-stroke statin (%)	7 (46.7%)	2 (18.2%)	*P =* 0.13
Pre-stroke antiplatelet (%)	5 (33.3%)	3 (27.3%)	*P =* 0.74
History of cardiovascular disease (%)	5 (33.3%)	3 (27.3%)	*P =* 0.74
Total cholesterol	4.55 (± 1.3)	4.5 (± 0.88)	*P =* 0.91
Median NIHSS (IQR)	5 (12)	4 (8)	*P =* 0.70
Thrombolysed	2 (13.3%)	4 (36.4%)	*P =* 0.17
Modal degree of symptomatic artery stenosis	70–89%	70–89%	
Maximum stenosis in symptomatic artery			
CCA	1 (6.7%)	1 (9.1%)	
ICA	14 (93.3%)	10 (90.9%)	*P =* 0.82
Modal degree of asymptomatic artery stenosis	30–49%	30–49%	
Maximum stenosis in asymptomatic artery:			
CCA	3 (20%)	2 (18.2%)	
ICA	12 (80%)	9 (81.8%)	*P* = 0.90
Mean onset-to-FDG-PET/CT (SD) (days)	9.2 (± 4.8)	8.9 (± 4.7)	*P =* 0.88

**Figure 1:**
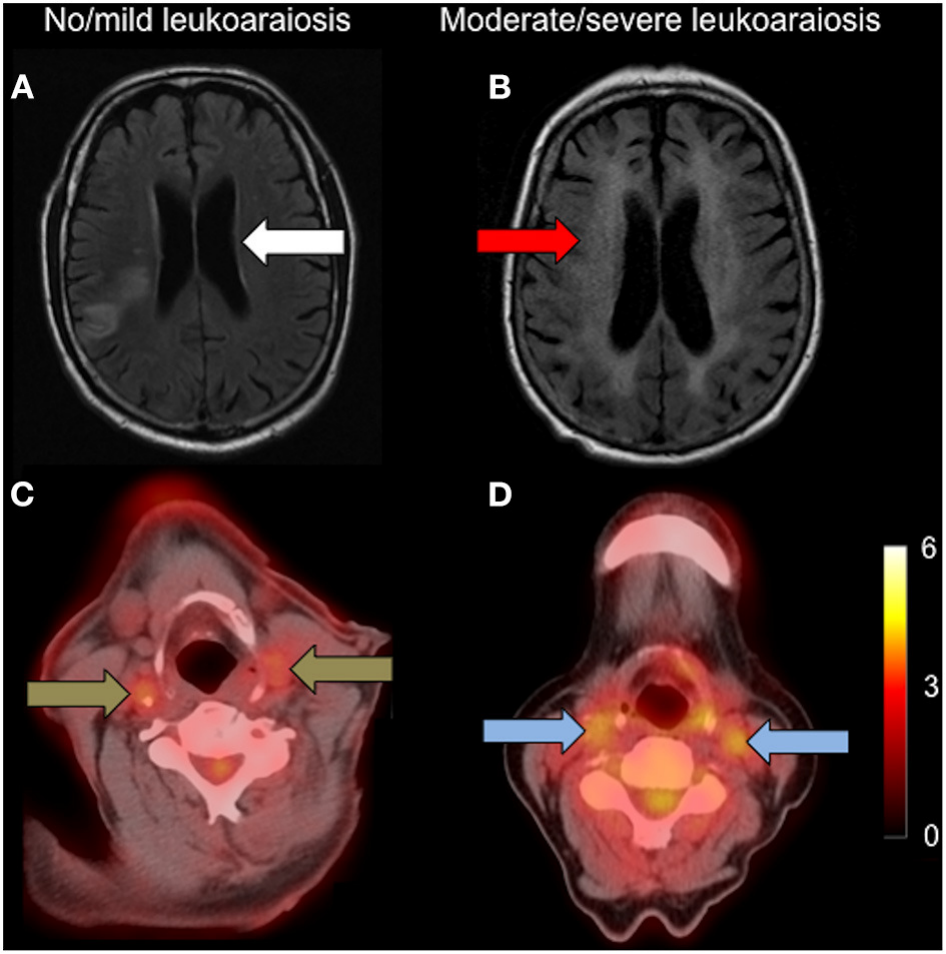
Carotid FDG uptake according to leukoaraiosis severity. Left: **(A)**: axial FLAIR showing no/mild leukoaraiosis (white arrow) with **(C)**: associated low carotid FDG uptake (brown arrows); Right: **(B)**: axial FLAIR showing moderate/severe leukoaraiosis (red arrow) with **(D)**: higher associated carotid FDG uptake (blue arrows). Both FDG-PET/CT images are set to the same scale, with the scale bar showing FDG SUV.

Multiple logistic regression showed FDG uptake to be independently associated with severity of leukoaraiosis, for both plaque and average whole vessel and in both culprit and contralateral non-culprit arteries (Table 3; Figure 1). The strongest associations were for the non-culprit artery, in particular the WV uptake [adjusted OR 6.18 (95% confidence interval 2.1–18.2), *P* < 0.01]. This model also suggests a lower odds of moderate/severe leukoaraiosis in individuals taking statins and increased odds of more severe small vessel disease with increasing age. The effects of diabetes and smoking were inconsistent (Table 3).

**Table 3:** Multiple logistic regression for moderate/severe leukoaraiosis severity for focal (MDS) and whole vessel (WV) FDG uptake in culprit and non-culprit carotid arteries.

**Culprit artery**			**Non-culprit artery**		
**MDS TBR_**MAX**_**		**Adjusted R^**2**^** **= 0.48 (*P < * 0.01)**	**MDS TBR_**MAX**_**		**Adjusted R^**2**^** **= 0.62 (*P < * 0.001)**
	**OR (95% CI)**	**Significance**		**OR (95% CI)**	**Significance**
FDG uptake	2.14 (1.07–4.28)	*P =* 0.04	FDG uptake	3.98 (1.84–8.59)	*P < * 0.01
Age	1.03 (1.01–1.05)	*P < * 0.01	Smoking	5.55 (1.23–25.0)	*P =* 0.04
Pre-stroke statin	0.64 (0.46–0.89)	*P =* 0.02	Age	1.03 (1.02–1.05)	*P < * 0.001
Smoking	2.96 (0.66–13.32)	*P =* 0.17	Pre-stroke statin	0.71 (0.54–0.93)	*P =* 0.02
**WV TBR**_**MAX**_		**Adjusted R** ^**2**^ **=** **0.49 (** ***P<*** **0.001)**	**WV TBR**_**MAX**_		**Adjusted R** ^**2**^ **=** **0.57 (** ***P<*** **0.001)**
	**OR (95% CI)**	**Significance**		**OR (95% CI)**	**Significance**
FDG uptake	1.52 (1.06–2.17)	*P =* 0.03	FDG uptake	6.18 (2.10–18.2)	*P < * 0.01
Age	1.03 (1.01–1.05)	*P < * 0.01	Age	1.03 (1.01–1.05)	*P < * 0.01
Pre-stroke statin	0.63 (0.46–0.86)	*P < * 0.01	Pre-stroke statin	0.72 (0.53–0.97)	*P =* 0.04
	Diabetes	2.15 (1.41–3.28)	*P < * 0.01
	Smoking	8.34 (1.14–61.0)	*P =* 0.05

*FDG uptake refers to per unit increase in the stipulated TBR_max_*.

Quantitative measures of WMH produced a similar pattern. On univariable analysis, there was no relationship between culprit carotid MDS or WV TBR_max_ (r_s_ = 0.30, *P* = 0.14 and r_s_ = 0.20, *P* = 0.34, respectively). In contrast, there was a trend of increasing strength of association between WMH volume with median non-culprit MDS TBR_max_ (r_s_ = 0.39, *P* = 0.05), and the WV TBR_max_ of the non-culprit carotid (r_s_ = 0.50, *P* = 0.01).

Linear regression of WMH volume, adjusting for cardiovascular risk factors, broadly supported the findings in the semi-quantitative analysis. Again, FDG TBR_max_ was independently associated with increased WMH volumes for diffuse measures of atheroma inflammation (non-culprit artery readings and the median whole vessel uptake in the culprit carotid), but not when considering the focal uptake in the culprit plaque (Table 4). Furthermore, this analysis also indicated a consistent independent positive association between age and WMH volume, and a negative association between statin use and WMH volume, in-keeping with the results observed in the semi-quantitative analysis. There were no significant interactions between these variables.

**Table 4:** Linear regression for white matter hyperintensity volume (mL) for focal (MDS) and whole vessel (WV) FDG uptake in culprit and non-culprit carotid arteries.

**Culprit artery**			**Non-culprit artery**		
**MDS TBR_**MAX**_**		**Adjusted R^**2**^ = 0.49** **(*P < * 0.01)**	**MDS TBR_**MAX**_**		**Adjusted R^**2**^ = 0.59** **(*P < * 0.001)**
	**Beta coefficient**	**Adjusted significance**		**Beta coefficient**	**Adjusted significance**
TBR_max_	3.53	*P =* 0.08	TBR_max_	9.38	*P < * 0.01
Age	0.50	*P < * 0.01	Age	0.50	*P < * 0.001
Pre-stroke statin	−12.6	*P < * 0.01	Pre-stroke statin	−9.03	*P =* 0.02
Pre-stroke antiplatelet	6.05	*P =* 0.15	Pre-stroke antiplatelet	5.69	*P =* 0.12
**WV TBR**_**MAX**_		**Adjusted R**^**2**^**=0.** (***P<*** **0.001)**	**WV TBR**_**MAX**_		**Adjusted R**^**2**^**=0.62** (***P<*** **0.001)**
	**Beta coefficient**	**Adjusted significance**		**Beta coefficient**	**Adjusted significance**
TBR_max_	8.91	*P < * 0.01	TBR_max_	14.33	*P < * 0.01
Age	0.52	*P < * 0.001	Age	0.48	*P < * 0.001
Pre-stroke statin	−11.54	*P < * 0.01	Pre-stroke statin	−8.86	*P =* 0.01
Pre-stroke antiplatelet	6.69	*P =* 0.07	Pre-stroke antiplatelet	6.33	*P =* 0.08

Inter-rater reproducibility of Fazekas scoring had an ICC of 0.91 across all scans. Inter-rater reproducibility of WMH volumes had an ICC of 0.99.

## Discussion

Our study is novel in relating the presence of leukoaraiosis to the physiological activity within systemic atherosclerosis measured using PET, rather than simply the degree of anatomical luminal stenosis. We demonstrate an independent association between atheroinflammation within carotid atherosclerosis and the severity of small vessel disease.

This relationship, and the strength of the regression models themselves, was stronger when considering the contralateral non-culprit artery rather than the culprit artery. The non-culprit artery is likely more representative of the overall burden of systemic atheroinflammation, in effect acting as a disease “barometer,” as suggested by the correlation between neighboring arterial regions demonstrated by Rudd et al. (23). In contrast, the most diseased segment of the culprit symptomatic artery represents a region with potentially disproportionate uptake – a peak focus of inflammation possibly accentuated by the rupture itself – that may not be reflective of the global burden of atheroinflammation throughout the body. Supporting this, our results indicate more diffuse measures of FDG uptake in the culprit artery (i.e., the WV) are similar to those from the non-culprit artery. Given that WMHs represent chronic disease developing over a longer time course than acute stroke, it is therefore likely that the non-culprit artery gives a better representation of the long-term pathophysiology to which the brain has been exposed.

A possible mechanism linking atheroinflammation and SVD is the action of matrix metalloproteinases (MMPs), which may act locally and systemically. A single vulnerable plaque may rupture through MMP-mediated disruption of the fibrous cap (1, 24), but the elevated plasma concentrations (particularly of MMP-9) seen in atherosclerosis may also have important systemic effects (25, 26). MMP-9 is implicated in blood-brain barrier dysfunction (27–29), where increased permeability may promote the development of leukoaraiosis (30, 31). Previous studies have demonstrated an association between FDG uptake and serum MMP-9 concentrations (23, 32). A 12-week course of atorvastatin 40 mg/day resulted in significant reductions in both atheroma TBR and MMP-9, with a moderate correlation between the reduction in plaque TBR and reduction in MMP-9 concentration (33). These relationships, and those between MMP-9 levels and blood-brain barrier dysfunction (27–29), and between blood-brain barrier permeability and the development of leukoaraiosis (30, 31, 34), indicate an association between the chronic atheroinflammation within carotid plaques and the development of leukoaraiosis.

Our finding that FDG uptake did not relate to the degree of luminal stenosis may explain the previously-reported inconsistent findings in the association between leukoaraiosis and the degree of stenosis (14), where plaques with similar degrees of stenosis may have different levels of atheroinflammation. The positive associations between SVD and increased IMT or presence of plaque are in-keeping with this hypothesis, as they may represent an earlier stage of atherogenesis (and one more associated with inflammation) than the degree of stenosis, where there may be more variability in plaque activity from highly inflamed early atheroma to older quiescent plaques.

Previous work has reported inconsistent associations between leukoaraiosis and a range of vascular risk factors (35). A notable exception is age, which most studies have found to be independently associated with the development of WMHs (13, 36). Our findings support this. Furthermore, the independent association of statin therapy with reduced SVD is potentially consistent with the pleiotropic effects of statins and hence relevant to the inflammatory hypothesis. The role of statins in WMH progression remains a subject of debate: in the PROSPER study there was no effect on WMH progression with pravastatin, though this cohort had low rates of atherosclerosis (37). In contrast, progression of confluent WMHs was found to be reduced by the use of pre-stroke statin therapy (38).

### Limitations and Future Work

Although the high sensitivity of PET enables detection of subtle physiological changes, allowing statistically significant differences to be detected despite small sample sizes, the limited size of our study means that further validation through replication in a larger cohort or meta-analysis would be advantageous.

Related to this, some caution must be exercised when interpreting the regression analyses given the relatively small sample size. The use of the Akaike information criteria in backwards elimination to optimize best-fit ensures that the selected models explain the greatest amount of variation using the fewest number of independent variables, hence reducing the risk of overadjustment bias. In our linear and logistic models, the consistent inclusion of age and pre-stroke statin in such optimized models is biologically plausible and supported by the existing literature as discussed above. Their presence is likely to be on the causal pathway, thereby reducing overadjustment bias further. Although the final models typically include three to four covariables for the study size of 26 participants, and hence not meet the “rule of ten” for the ratio of outcomes to variables, such a rule of thumb has been argued to be either too conservative or potentially of limited evidence basis (39, 40). However, further replication and validation in larger studies to accommodate more variables will be advantageous to reduce further the risk of overadjustment bias.

We did not measure MMPs in this study, though the association between FDG uptake and MMP-9 has been reported previously (23). Future studies measuring MMPs and other inflammatory biomarkers may further elucidate the mechanistic link underlying associations observed here.

In this study, we considered only carotid atherosclerosis. The overall burden of systemic atheroinflammation will reflect the totality of disease in other arterial territories (including coronary arteries, aorta, and peripheral arterial disease). However, previous work has demonstrated that atheroinflammation is strongly associated across neighboring arterial territories, and consequently the carotids (particularly the diffuse measure of uptake in the non-culprit artery, WV TBR_max_) may serve as good surrogates of systemic atheroinflammation (23). Furthermore, we found a moderate association between the non-culprit WV TBR_max_ and serum hsCRP, suggesting that the carotid uptake is a reasonable reflection of systemic inflammation. Future work considering the global burden of atheroinflammation for the individual, incorporating atheroinflammation across coronary, aortic, and peripheral arterial disease, as well as comparison against healthy controls would help elucidate this relationship further.

Although highly sensitive, FDG uptake is non-specific. Although the measures taken here improve its specificity for inflammation, replication using newer radiotracers with higher specificity for inflammatory cells, such as ^68^Ga-DOTATATE (19), would help characterize this relationship.

To elucidate the mechanisms underlying the associations observed in this study, future work should consider a range of biomarkers of systemic inflammation, and imaging of BBB integrity alongside carotid and brain imaging.

## Conclusion

The observed association between carotid atheroinflammation and the presence of more severe small vessel disease has implications for our understanding of the neurovascular interface and may have future influence on how we manage “asymptomatic” atherosclerosis, with atheroinflammation treated more aggressively with anti-inflammatory agents. Canakinumab (a monoclonal antibody targeting interleukin-1β) has shown promise for reducing cardiovascular outcomes after myocardial infarction (41), whilst colchicine has also been found to reduce cardiovascular outcomes in those with coronary artery disease (42, 43). Evidence for the benefit of such agents related specifically to carotid atherosclerosis is currently lacking (44), though the Colchicine for Prevention of Vascular Inflammation in Non-cardio Embolic Stroke (CONVINCE) study will consider the use of colchicine in a stroke setting. Such therapeutic approaches targeting systemic atheroinflammation may have an important role for reducing the burden of chronic small vessel disease and its clinical sequelae.

## Data Availability Statement

The raw data supporting the conclusions of this article will be made available by the authors, without undue reservation.

## Ethics Statement

The studies involving human participants were reviewed and approved by Nottingham One Research Ethics Committee, 14/EM/0128. The patients/participants provided their written informed consent to participate in this study.

## Author Contributions

NE, AP, MG, JR, and EW participated in study design. NE, JT, JW, MC, and AP participated in data acquisition and analysis. NE performed the statistical analysis and drafted the manuscript. All authors participated in interpretation of the data and critical revision of the manuscript. All authors contributed to the article and approved the submitted version.

## Conflict of Interest

The authors declare that the research was conducted in the absence of any commercial or financial relationships that could be construed as a potential conflict of interest.

## Publisher's Note

All claims expressed in this article are solely those of the authors and do not necessarily represent those of their affiliated organizations, or those of the publisher, the editors and the reviewers. Any product that may be evaluated in this article, or claim that may be made by its manufacturer, is not guaranteed or endorsed by the publisher.
